# Data Hazards as An Ethical Toolkit for Neuroscience

**DOI:** 10.1007/s12152-024-09580-3

**Published:** 2025-02-19

**Authors:** Susana Román García, Ceilidh Welsh, Nina H. Di Cara, David C. Sterratt, Nicola Romanò, Melanie I. Stefan

**Affiliations:** 1 Centre for Discovery Brain Sciences, College of Medicine & Veterinary Medicine, Biomedical Sciences, University of Edinburgh, Edinburgh, UK; 2 Department of Oncology, University of Cambridge, Cambridge, UK; 3 School of Psychological Science, University of Bristol, Bristol, UK; 4 Institute for Adaptive and Neural Computation, School of Informatics, University of Edinburgh, Edinburgh, UK; 5 Zhejiang University-University of Edinburgh Joint Institute, Zhejiang University School of Medicine, Haining, PRC China; 6 Department of Medicine, Medical School Berlin, Berlin, DE Germany

**Keywords:** Data Hazards, Ethical labels, Ethical toolkit

## Abstract

The Data Hazards framework (Zelenka, Di Cara, & Contributors, 2024) is intended to encourage thinking about the ethical implications of data science projects. It takes the form of community-designed data hazard labels, similar to warning labels on chemicals, that can encourage reflection and discussion on what ethical risks are associated with a project and how they can be mitigated. In this article, we explain how the Data Hazards framework can apply to neuroscience. We demonstrate how the hazard labels can be applied to one of our own projects, on the computational modelling of postsynaptic mechanisms.

**Graphical Abstract:**

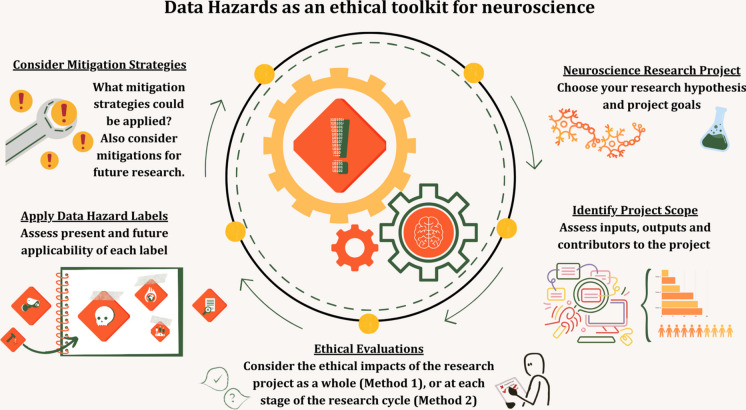

**Supplementary Information:**

The online version contains supplementary material available at 10.1007/s12152-024-09580-3.

## Introduction: The Need for a Practical Assessment Tool for the Ethical Impacts of Neuroscientific Work

To understand the social and ethical impacts of neuroscientific work, it is crucial that neuroscientists understand the wider context of their research and its possible impacts. However, they do not always have the relevant training, with only about half of neuroscience degree programmes offering formal ethics training as part of their curriculum [1].

A number of ethical frameworks and tools are available, but they often focus on particular questions. For instance, research on human participants and non-human animals (henceforth, animals) is governed by institutional and governmental regulations that cover only a limited number of the ethical considerations that neuroscientists may have in this area [2]. There are proposals by researchers and research councils for ethical frameworks in various realms of science including citizen science and patient participation [3], presentation of results to policymakers [4], Artificial Intelligence (AI) in education [5], and research integrity [6], Museum Group, [7]). Some research funding councils have mandatory frameworks for research ethics [8], but see Stanley & Wise [9] for a critique.

We believe that neuroscience could benefit from a modular, community-driven and adaptable ethics framework that can accommodate a broad range of research scenarios and use cases, and that encourages deep holistic reflection on the ethical implications of a particular research project.

Recently, the Data Hazards framework [10] has been developed to provide such a tool in the field of data science. In order to make the ethical risks of research tangible, it assigns labels, similar to chemical hazard labels, to specific risks such as “danger of misuse” or “high environmental impact”. Researchers using the framework can identify risks that apply to their projects and consider which safety precautions could be implemented to mitigate these before, during and after a data science research project. The Data Hazards framework is an open-source, community-driven project that follows FAIR principles (Findable, Accessible, Interoperable, and Reusable work [11]). The developers of the framework look for ongoing collaborative feedback from different disciplines so that it can keep up with rapidly developing areas where new ethical concerns are frequently appearing.

We propose that Data Hazards can be extended to serve as a useful toolkit for ethical reflection in neuroscience. In this paper, we first describe examples of how the Data Hazards labels may apply in neuroscience research and discuss what mitigation strategies might be considered for each of the labels. We then offer a step-by-step explanation of how to use the framework and demonstrate it using the computational neuroscience PhD research of the first author as an example. Finally, we propose two new Hazard labels that increase the applicability of Data Hazards to neuroscience.

## Data Hazards as a Philosophical and Practical Toolkit for Neuroscience

### The Data Hazards Project

The Data Hazards Project [10] has created a community-driven interdisciplinary vocabulary of ethical risks. The project presents these risks through Data Hazard labels, which look similar to chemical hazard labels. The labels can be used to foster interdisciplinary discussions about data ethics, and support self-reflection on various data ethics issues. Currently the project is on Version 1.0, which contains eleven Data Hazards [12, 13]. The project is open-source, aims to follow FAIR principles [11], and invites ongoing contributions to refine and develop the labels for future versions in order to keep up with changing ethical risks.

Each label is presented with descriptions, examples and safety precautions or mitigations that give suggestions on how to address these risks. The labels themselves are available at https://datahazards.com/labels. Even though the Data Hazards framework was originally created with data science in mind, and with a more AI and computational foundational rather than scientific focus, we argue that it can serve as a philosophical and practical toolkit that brings ethical considerations to the forefront of scientific inquiry across many fields, including neuroscience. By incorporating relevant Data Hazard labels into research outputs, scientists can communicate not only their findings but also the ethical considerations that underpin their work. This transparency invites a broader dialogue within the scientific community and beyond, fostering a culture of responsible and reflective scientific practice.

### Data Hazards in Neuroscience

Firstly, we illustrate how the Data Hazards framework applies to neuroscience by discussing the label “Reinforces Existing Biases” as an example. We provide examples for all the Data Hazard labels in Table 1. The label “Reinforces Existing Biases” highlights the potential for perpetuating unfair treatment towards certain individuals or groups. There is an extensive history of how neuroscience has helped advance scientific ableist and racist bias, from ableist IQ tests to pseudoscientific ideas about smaller skull sizes in so-called “inferior” races [55, 56]. In the context of scientific racism in neuroscience, there is also extensive critique of the historical (and current) overpathologizing, misdiagnosis and dismissal of valid symptom reports in people of colour’s health [57–60]. With the advent of technologies that use existing datasets for automated decision making, there is a risk of reproducing those pre-existing biases [16, 17]. There is overlap here with other Data Hazard labels, such as “Ranks or Classifies People”, “Lacks Community Involvement” and “Automates Decision Making”. Mitigation strategies to prevent and move on from the danger of reinforcing existing biases may include (but are not restricted to) careful assessment of training data, and the inclusion of a variety of viewpoints, for instance by involving the communities the research is designed to serve in the planning and decision-making.

**Table 1:** Neuroscience specific examples of Data Hazards

Image	Data Hazard Label	Neuroscience-specific Examples	Suggested Safety Precautions
General Data Hazard	The work uses data, and so should be interpreted with an understanding that all data has inherent drawbacks. The responsibility for using any outputs or conclusions ethically lies with those implementing them.	Many types of data and information are collected and used in the field of neuroscience, including the use of big data datasets [14].	When using data to back up scientific claims we should always be clear about limitations and assumptions made in the generation of the data, the results, and the conclusions of our work. This can be supported by conducting research in a transparent and reproducible manner [15], and that data is shared FAIR-ly [11] with all the necessary meta-data for it to be understood.
Reinforces Existing Biases	There is a risk of reinforcing unfair treatment of individuals or groups due to biases present in input data, algorithm design choices, possibly stemming from societal biases.	Issues related to the use of biased algorithms or data sources have been highlighted by different studies [16, 17]. For example, brain scans can be used to identify specific conditions, or facial recognition can be used to analyse facial expressions and emotional responses. However, if the data used to train the algorithm over-represents specific populations, the algorithm might reach biased conclusions, with the risk of misdiagnosing individuals from underrepresented groups.	Algorithmic bias can be mitigated by ensuring that the training data is diverse; implementing techniques to detect and measure bias and bias mitigation methods, such as data augmentation or adversarial training [18, 19]; pushing for the creation of more explainable models [20]; and ensuring continuous monitoring of the algorithm’s performance and bias.
Ranks or Classifies People	Classifies people in ways that can be arbitrary, unfair, or discriminatory and can result in harm to individuals or groups.	Classification of people as epileptic only based on “abnormal” EEG can lead to high false positive rates and can result in unnecessary treatment and suffering for the patients [21]. Use of ill-defined criteria for racialized classification of individuals in genetic studies, in neuroscience and other fields, results in dichotomization of what is a continuous range of genetic landscapes and can also create or reinforce societal biases [22].	Classification and rankings should be carefully contextualized within the scope of the study; extreme care should be taken in the interpretation and generalization of these results. Limits in methodologies should be acknowledged and discussed, and classification based on a single source of data should be avoided.
High Environmental Impact	Risk of energy-intensive algorithms, and/or work which requires special hardware, which can have a negative environmental impact.	Computational-heavy algorithms can require a large amount of energy and specialized hardware. This is particularly obvious in, but not limited to, the case of training large deep learning models. For example, the training of the AlphaFold2 method for prediction of protein folding required 128 TPUv3 cores over a few weeks [23]. A single day of training on this hardware generates 93 Kg CO_2_e [24].	Environmental impact of computational research can be limited by using more energy-efficient algorithms and hardware; using open-source software and hardware can also help reduce the environmental impact of research by reducing the need for duplication of resources. Wet-lab research can reduce its environmental impact by implementing green lab practices, switching to alternative materials and improving waste management; raising awareness and providing education to researchers with respect to the impact of their research on the environment is also a key mitigation strategy for the field at large.
Lacks Community Involvement	The project aims to serve a community, but members of that community have had little to no input in the design and implementation.	Wearable robotics designed without user input can result in products that are impractical, burdensome, or expensive to use (reviewed in [25]). Therapy approaches may lack involvement from the community it aims to help. For example, the “Applied Behavioral Analysis” (ABA) used in psychiatry has faced recent critiques for promoting dependence and creating a sense of shame around aspects of the Autism Spectrum, encouraging individuals to be more “neurotypical” [26–28].	Some proposed guidelines for the design of wearable robotics implicitly include the necessity of input from relevant stakeholders such as users and their families [29], who should be ideally involved from the planning stage, or at least in later stages of ongoing projects. Literature research should not only include the scientific perspective on the subject, but also take into account how relevant communities are impacted (see [30] for patient engagement strategies in neuroscience). An example of community involvement can take the shape of including patients and their families in research on neurological diseases. For instance, the Chan-Zuckerberg foundation put out a funding call in 2022 for patient-partnered projects for neurodegenerative diseases [31].
Danger of Misuse	Some method or technology used in research can be misused or used for malicious purposes.	Consumer-grade EEG devices pose a series of ethical issues, often overlooked by bold marketing claims [32]; they are also prone to malicious attacks which could be used to extract sensitive information from users [33].	Implementing safety protocols to avoid misuse of technology (e.g., encryption and anonymization of data, avoiding storage of unnecessary data that is at risk of being stolen); raising awareness of potential misuse and review any commercial claims of the technology. Restrictions on further use of technology to ethical purposes only have been discussed and occasionally implemented in the software realm [34], but there is debate on the usefulness of such an approach [35].
Difficult to Understand	Projects are often difficult to understand, either because the subject matter itself is complicated or opaque, or because of issues with project implementation, for instance a lack of transparency about the methods and underlying data, or insufficient documentation.	Investigating the cellular and molecular basis of brain function requires advanced knowledge of biology. Underlying concepts, e.g., of genetics, are not well understood by the general public (for instance, [36]), thus making such research challenging to understand for most people. In a study on 455 models in computational systems biology, (49%) were not directly reproducible, mostly because of missing information on parameter values and initial conditions [37].	Thoughtful science communication strategies [38] and inclusion of stakeholders throughout all stages of the research cycle (see above) can help make our research accessible to a broader audience. Methodological transparency can be improved by sharing data and code using the FAIR principles [11].
May Cause Direct Harm	The project or its direct outcomes can cause direct harm, even if used as intended.	A lot of neuroscience research still relies on animal models. For instance, Keifer and Summers [39] estimate that in 2015, (32%) of all published neuroscience articles were on work done on rodents alone. There are many historical examples of unethical research on human subjects, causing great physical and psychological harm (reviewed in [40, 41]).	Use the 3Rs [42], cited in [43] to refine, reduce and replace animal research (but see also [44]). When working with human participants, communicate clearly about risks of potential harm. Conduct a thorough and thoughtful risk assessment. Monitor ongoing experiments and have clear criteria for when to abort.
Risk to Privacy	The project involves the use of personal data (e.g., patient data) and the privacy of the people whose data is used or of their families is put at risk.	De-identified data can, in principle, be re-identified. In theory, anonymization is stronger than de-identification because it is meant to be irreversible (reviewed in [45]). However, even data thought to be anonymized may be traceable to individuals using newer technologies. For instance, a recent study was able to match anonymized MRI data to individuals using facial recognition software [46]. Another example is testing for some genetic conditions, where testing for a particular allele may also reveal the carrier status of a family member who did not consent to being tested (reviewed in [47]).	Good institutional data storage and handling policies should be in place to avoid involuntary data leaks. If robust anonymization cannot be guaranteed, it may be better not to share raw data. We recognize that there is tension between principles of open science and transparency and safeguarding participants’ privacy, also noted in [45]. Have protocols for how to handle implicit genetic information about family members.
Automates Decision Making	This research, or a product thereof, can be used to automate decision-making.	There is an increasing body of work on using machine learning for diagnosis (e.g., [48]) or classification (e.g., [49]) of neurological conditions. While still in its early days, it is conceivable that such algorithms may be used to at least partially automate clinical and non-clinical (e.g. for insurance purposes) decision-making.	Clearly communicate the assumptions that go into an algorithm, the uncertainty associated with a prediction, and what it means. Suggest best practice procedures for machine-assisted diagnosis, clarifying where and how human input is needed [50].
Lacks Informed Consent	The project involves the collection of data, samples, or relies on previously collected data which were obtained without the informed consent of participants.	Anything done on HeLa cells, as they were originally obtained and developed without the explicit consent of Henrietta Lacks. HeLa cells have been employed in over 110,000 scientific publications to investigate the impact of different drugs, hormones, different cancer research methods, and even taken into space [51, 52]. There have been countless ethical debates and reforms surrounding patient consent since the details of the story were published, and other examples of cell lines used without prior consent [53, 54].	Good institutional data storage and handling policies should be in place to avoid involuntary data leaks. If robust anonymization cannot be guaranteed, it may be better not to share raw data. We recognize that there is tension between principles of open science and transparency and safeguarding participants’ privacy, also noted in [45]. Have protocols for how to handle implicit genetic information about family members.

Another widespread bias in neuroscience is speciesist bias. There are arguments for the importance of animal models, for instance in the study of memory [61–65]. Much of our current knowledge and understanding of neuroscientific questions have been developed through experiments involving the use of animals. Without these experiments and their findings, our understanding of significant areas of neuroscience might be considerably less. However, experiments on animals rely on anthropocentric views where human health is prioritized over non-human health. Further, some animal species are widely considered more morally acceptable research subjects than others, depending on factors such as their phylogenetic proximity to humans, their perceived intelligence, or whether or not they are commonly held pets (reviewed in [66]). There is often a utilitarian harm-benefit argument made to justify animal usage in relation to potential human benefits [67], but even researchers who subscribe to this utilitarian argument may consider acknowledging the underlying assumptions if prompted with the Reinforces Existing Biases label.

Like other disciplines, neuroscience is affected by citation count bias. Citation counts influence how likely we are to see an article on search platforms such as Google Scholar [68]. Even without this algorithmic bias, there is evidence for a “Matthew effect” in science, whereby already successful scientists or institutions are given more resources, opportunities, and attention, thus making them even more visible [69]. Even if we personally were completely free from any kind of prejudice or bias (which is highly unlikely [70]), the existence of these and other biases within science means that some research outputs are more likely to reach us than others, and our work therefore stands on biased foundations. Citation-count bias also interacts with other biases, such as systemic racism: Non-white scientists are likely to be cited less frequently, be underrepresented on editorial boards and have higher waiting times before publication [71]. Addressing these issues as an individual is not enough, which emphasizes the importance of collaboration at various levels for effective mitigation.

This is not meant to be an exhaustive discussion, but more of an illustration of what this particular Data Hazards label means to us. We hope that readers can use this to support their own reflective process. In Table 1, we briefly discuss all eleven Data Hazards with examples from neuroscience and possible mitigation strategies.

Recognising that neuroscientific research has far-reaching consequences for individuals and society requires a deeper inquiry into the ethical dimensions of how we use data in neuroscience. The labels allow researchers to grapple with questions of bias, privacy, and the potential societal impact of their findings.

## How to use the Data Hazards Project

The Data Hazard labels can be used in different settings, making it a valuable toolkit for diverse applications and contexts. For example, the shared vocabulary of Data Hazard labels could be taught in a classroom setting, or as a conference workshop to a wide audience of scientists (see [72] for examples of workshops). This is a more didactic approach, where the labels are taught to a broad audience with case study examples, showing how the labels apply to different projects. This tool can also be used individually, where labels are applied to one’s own work. Ideally this toolkit would be first applied when planning a project, then iteratively re-used throughout, and as a final self-reflection.

The original authors of the Data Hazard project suggest applying the framework in a *Ethics by Design* manner (similar to *Privacy by Design*) [12, 73, 74], in which research ethics committees and researchers work together during all stages of a project, from initial design and conception through to undertaking the study and sharing results. This approach ensures that ethical considerations are integrated in the science, as opposed to being viewed as supplementary measures afterwards.

In line with this approach, one of the primary objectives of the Data Hazards framework is to encourage feedback from others on the ethics of a project, as opposed to only self-assessments. When it comes to showcasing and sharing the labels of a project, there is no one prescribed way to do this. We propose the use of a table provided in Supplementary Materials that could be added at the beginning of a research paper, or at the end of it too (see the following examples of different ways in which the labels have been applied to various projects [75–77]).

The interdisciplinary design of the labels means they can easily be used by experts from different disciplines to engage in discussions on these issues; for example, neuroscientists talking with ethicists and data scientists. Additionally, it is important to note that views on which labels may apply to a project can vary with time and differ between individuals. We see this as a strength of this framework, as it allows for continuous critique and conversation resulting in more nuanced and collaborative ethical considerations. Below we outline two suggested methods for applying the Data Hazards labels to neuroscience research.

### Step one: Outline the Project’s Scope

Ideally, before applying labels an overview of the project should be written. The writing process helps the researcher to gain a clearer view of how, where and why a label may apply in their research and helps with reflecting later on. This could be done using an existing project template (such as [78]) or in a free-form format.

Some prompts that can be used to think about this are:

- Project question/hypothesis
- Project objectives
- Type of data used, variables in the dataset, including what is included and excluded
- How and when was data collected, and by whom?
- Statistical/algorithmic methods used
- Who are the project contributors?
- Expected outputs and sharing strategies
- How is the research funded?

### Step two: Apply the Labels

We suggest two ways to proceed next. *Method 1*: examine the labels individually, one by one; *Method 2*: assess the labels across the entire research life cycle. These two methods are not mutually exclusive, as we will see in our application below, they can and do work well if used together, but can also be applied separately depending on the researcher’s needs.

- *Method 1: Apply labels one by one*

Researchers can go through each label, assessing whether it applies to the project, and if so, what precautions can be taken. Researchers may use following prompts to reflect through each label:

- **Present applicability.** Does the Hazard label apply currently to my project? If so, how and why is this? If not, why not?
- **Future applicability.** Is there a potential that this Hazard label does not apply currently, but will in the future?
- **Mitigation strategies.** What mitigation strategies could be applied? If it is not possible to mitigate the Hazard in this project, then devise mitigations for future research.

Some risks may not be preventable, but it is still important to highlight them in order to either develop future mitigation practices or at least create awareness of the problem.

An example of applying Method 1 in practice is shown as a case study further in this paper (see Method 1: Applying labels one by one to the PhD project).

- *Method 2: Apply Labels Through Research Life Cycle*

Here, researchers consider how and where the labels apply in the stages of the research cycle, for example through design, data collection, data analysis and reporting. This approach facilitates nuanced insights into when and where specific ethical considerations become pertinent in the research process. Moreover, future researchers and stakeholders, including those beyond the research community, benefit from this clarity as it enhances transparency and accountability in the research process. This comprehensive assessment ensures that ethical considerations are not an afterthought but an integral part of the entire research endeavour.

There is a template table in the Supplementary Materials. In the case study section of this paper, we provide an example of applying the labels in the research life cycle of a computational neuroscience project (see Method 2: How do the Labels Apply Through the PhD Project Research Life Cycle?).

#### Additional step: Propose New Data Hazard labels

The Data Hazard labels were developed to allow a shared vocabulary to overcome disciplinary silos and have constructive feedback about project ethics from people in different disciplines [10]. Therefore, in addition to applying the labels, one can contribute to the project too [10]. To this end, we propose and discuss two new Data Hazard labels in the case study section below.

## A case study: Applying the Data Hazards to a Computational Neuroscience PhD

Following the steps described, we will demonstrate how the Data Hazards framework can be applied to a computational neuroscience modelling project using the first author’s PhD research as an example.

### Step one: PhD’s Project Scope

#### Project’s Biological Question:

How does the interaction between Calcium/calmodulin-dependent protein kinase II (CaMKII) and N-methyl-D-aspartate receptor (NMDARs) in the postsynaptic neuron affect the function of both molecules? Changes in the function of these synaptic proteins have been implicated in a range of diseases and conditions, including age-related cognitive decline (see, for instance, (Li, Marcu, et al*.*, [79]) and neurodegenerative diseases (reviewed in [80, 81]). Moreover, these changes have been shown to be implicated in epilepsy, schizophrenia, addiction, autism spectrum condition, and multiple neurodevelopmental disorders (reviewed in [82]).

#### Project Objectives:

We employ computer simulations to answer the project’s biological question; it is important to note that while our focus is mainly computational, this is part of a synergistic work with colleagues performing wet-lab experiments. Experiments provide data to inform simulation parameters, which in turn can guide the wet-lab process by narrowing the experimental search space, in a self-enhancing loop. In short, the main objectives of the project are to:

- Build a computational model of the regulation and interactions between CaMKII and NMDARs in a dendritic spine.
- Make biological predictions from the model which can then be tested experimentally *in-vivo* or *in-vitro*.
- Use the model to simulate spatio-temporal dynamics of early LTP and understand the role of CaMKII/NMDAR interactions.
- Develop new workflows to model biological interactions in 3D space, using software like MCell (Monte Carlo Cell) and CellBlender [83–86], BioNetGen [87] and BioDynaMo [88].

#### Data Informing Model Parameters and Output data:

In order to understand the type of data used and variables in the dataset, we first explain what kind of data goes “into the model” (model parameters) and then what data “comes out” (model outputs). The data informing model parameters are kinetic rates, equilibrium constants of molecular interactions, cellular volumes and molecular concentrations. These parameters are used to create a set of reaction rules that determine how molecules behave. Based on these rules and parameters, a simulation is run that tracks the movement and interaction of molecules over time. The simulation generates time-course data that tracks concentrations, activity patterns and the spatial distribution of molecules. These data can be represented graphically as plots of concentrations against time, or as videos showing how molecules move through space over the course of the simulation.

#### How and When was Data Collected, and by Whom?

The PhD project is based on data previously published, and data for the project is manually researched and collated from the literature primarily by the PhD student, throughout the time course of the four-year project. The data collected relies on studies done both *in-vitro*, *in-vivo* and *in-silico*, which have published dendritic volumes, molecule concentrations [89] and proposed kinetics of CaMKII and NMDARs, along with other molecules that interact with them [79, 90–94]. Experimental values for kinetic rates and equilibrium constants are difficult to obtain with certainty,if no parameters can be found, they are estimated according to available data for the current model.

Most of the data used to parameterise and test our computational model come directly from previous studies using animals (for example [89, 94–97]), parameter databases that rely at least partially on studies done on animals [98, 99], or from previous computational models that have also been parameterised and validated using animal data (e.g. [79, 90–93, 100, 101]). Some studies were done using knockouts of certain protein genes to study their effect on memory impairment others with other methods such as targeted mutagenesis. They may involve invasive or distressing procedures on rodents and require animals to be sacrificed.

#### Software and Statistical/Algorithmic Methods Used:

Free, open-source software and algorithms are used to create 3D models of the molecular interactions in question.

- **MCell4** is a biochemistry simulation tool to model movements and interactions of discrete molecules within and between cells. Additionally, CellBlender release 4.0 is used as a plug-in for the 3D modelling software Blender 2.93 [102], which provides a graphical user interface for working with and visualizing MCell4 models [83].
- **BioNetGen (release 2.8.5)** is a set of software tools that enable modelling biochemical reaction kinetics and protein–protein interactions through a set of rules. It facilitates the modelling of multimeric protein structures such as that of CaMKII [87]. Together, MCell and BioNetGen make it possible to model CaMKII as a dodecamer that interacts with NMDARs and other molecules in a 3D simulation of a dendritic spine.
- **BioDynaMo (release 1.04)** is an agent-based modelling tool which can model 3D biophysical molecular interactions [88]. In this project, BioDynaMo provides the capability to simulate neuronal growth through formation of an actin cytoskeleton inside dendritic spines, a process which CaMKII has been shown to be involved with [103–105].

#### Who are the project contributors?

The main content and discussion contributions for the project are from the PhD student and her supervisors. Additionally, Masters and Undergraduate students have made valuable contributions as their dissertation thesis research addressed specific queries relevant to this PhD [106, 107]. Moreover, the Alan Turing Institute community played a crucial role in contributing to discussions on ethics and reproducibility of this PhD project. Likewise, the MCell, BioNetGen and BioDynaMo software development teams have been key for their contribution to bug fixing, coding and scripts on the simulation models. The broader scientific community and researchers contributing knowledge to the field of this PhD are indirect contributors, laying the foundations for this research.

#### Expected Outputs and Sharing Strategies:

The anticipated outputs of this research include predictions of the interactions of CaMKII and NMDARs along with other molecules in time and space, providing insights for future hypothesis testing by experimentalists. Following FAIR principles, the project aims to ensure that data and models are Findable, Accessible, Interoperable, and Reusable. A reproducible model will be shared on GitHub and mathematical model repositories, along with associated materials, which will enable others to validate findings and reuse the data for their own research. The documentation will encompass the settings for running the model and the generation process of figures so that future researchers can easily build upon this work. The results of this work will be published in open-access journals to ensure that the research is freely accessible to the public, promoting broader dissemination and impact. The PhD author may submit negative findings to the Journal of Trial and Error [108] or bioRxiv [109].

### Step two: Ethical Evaluation of the Project: Applying the Labels

After discussing the labels more in-depth using Method 1, we will reflect on where in the research life cycle they may apply using Method 2.

#### Method 1: Applying Labels One by One to the PhD Project

Each of the labels is interpreted as having high, medium, or low relevance to the PhD project case study. Reflections and examples are offered on how they are more or less relevant, and mitigation and safety precautions are proposed, either already taken into action or for future researchers to consider.

##### General Data Hazard - High relevance.

The General Data Hazard is a universal reminder that ethical considerations apply to all projects which involve data. Therefore, it applies to this project. The purpose of this label is to emphasize individual and collective responsibility for a project, and to encourage reflection about its current and future uses and implications. It is equally important to also consider mitigation strategies that can be applied now or in the future.

##### Reinforces Existing Biases - High Relevance.

The authors of this paper see this label not only applying to methods of measuring, collecting or reporting data, but also to individuals or groups. How a project modelling molecular interactions in the synapse might reinforce existing biases may not be immediately obvious, but upon reflection, we identified a few areas where this work may reinforce already existing biases.

Ableist bias. As researchers in synaptic neuroscience, we frequently use diseases affecting the synapse as a way to motivate our research to students, audiences, and funders. By using neurological conditions as a motivation for our research, we risk perpetuating the narrative that they are all medical problems to be “solved” or “cured”. To reduce perpetuation of this bias, we acknowledge our research’s position within a scientific culture that prioritizes “fixing” under a medical model of disability. Mitigation for ableism involves addressing systemic issues by fostering inclusivity in the workforce, avoiding tokenism or mere rhetoric. For example, researchers involved in this work should consider their own identity and positionality, power and privilege. The Turing Way Booklet offers a self reflection chapter with useful prompts for this purpose such as *“Who isn’t included in the data I have collected or reusing an existing dataset?”* and *“What were the dominant beliefs at the time?”* [15].

Positive-results bias. There is a tendency for studies to emphasize novel or positive outcomes in scientific research [110]. This type of bias can create a distorted view of scientific knowledge. Researchers, readers, and policymakers may be misled by an incomplete and overly optimistic representation of the evidence, leading to misguided decisions, wasted resources, and potential harm in fields like medicine and public policy. To address this bias in this PhD, a strategy taken is to ensure model reproducibility. Transparent methodology, rigorous peer review, cross and external validation, and conducting sensitivity analysis also serve as effective mitigations. Additionally, supporting platforms like the Journal of Trial and Error can contribute to overcoming this bias by providing space for publishing results that deviate from the expected positive outcomes.

##### Ranks or Classifies People - Low relevance.

This project doesn’t directly rank or classify people. However, future work based on this work could be used to classify patients. For example, “normal” or “typical” behaviour versus “abnormal” or “divergent” (see also ableist bias in previous label). Classifying individuals is not necessarily a bad thing, but it can be if it benefits some individuals over others. While classification may be necessary for diagnosis, reflecting on the intent and implications of classification becomes crucial. Since this label has low relevance for the PhD project at the moment, mitigations and safety precaution measures will become more relevant with future work. Future safety precautions could be integrating interdisciplinary and inclusive teams that would scrutinize the criteria used for classification, ensuring fairness, inclusivity, and avoiding the reinforcement of existing biases.

##### High Environmental Impact - Medium relevance.

To estimate the environmental impact of running the models in this PhD, we estimate the energy consumed by the computer hardware and associated cooling, and convert it to CO_2_e emissions using the Green Algorithms Calculator v2.2 [111]. For example, one simulation run of a model in the PhD project on cloud computing (a University of Edinburgh computer cluster) takes 4 h on 1 CPU Xeon E5-2665, using 2 GB of memory, and is estimated to consume 0.101 kWh. In the UK, this has a carbon footprint of 23.4 g CO_2_e, which is similar to other common research activities, such as 15–150 g CO_2_e for a 1 h Zoom call [111], or 21 g CO_2_e to make a cup of black tea [112]. The environmental impact increases with the number of simulations, and it is likely that hundreds or thousands of simulations will be required during the PhD project. Running the model 30 times with the same requirements would consume 3.03 kWh, equivalent to a 4 km car ride.

Therefore, although the current environmental impact of this PhD is relatively low, models created during the PhD project could have a high environmental impact in the future as they grow in complexity. Environmental impact not only includes energy consumption by the computer hardware as we have described; there is also the life cycle impacts of manufacturing, maintaining and disposing of hardware which are more difficult to calculate [113]. For these reasons, we see the relevance of this label to the PhD project as medium.

In general, mitigations to reduce the environmental impact of computational projects include optimizing computational workflows to minimize energy consumption and considering carefully which simulations are necessary. It may be possible to choose energy-efficient computing resources, for example by using servers located in countries with lower carbon footprints of electricity consumption. Even if these mitigations are not feasible at present, the process of reflection can help to develop strategies to reduce environmental impacts in future projects.

##### Lacks Community Involvement - Low Relevance.

While not an immediate concern, this label is expected to become more pertinent in the future. We can see how community involvement for this label applies to different groups. On the one hand, there is the scientific community engaged in research discussions. Prioritizing reproducibility, thorough documentation, and the dissemination of results from the project’s start significantly contributes to mitigating this specific hazard. On the other hand, the community comprising families and patients affected by research outcomes, is not a major focus presently but requires future attention. Future recognition towards patient community involvement could include using platforms for open dialogue between researchers and patients, seeking input on research priorities, questions and methodologies [114, 115], and utilizing existing community involvement frameworks [116–118], which we further explore in the discussion section of this paper.

##### Danger of Misuse - Medium relevance.

The authors of this article can see various ways in which danger of misuse could apply to the PhD project. Firstly, due to misunderstanding (see Difficult to Understand) or use of faulty results (see related Proposed new label: Potential of Faulty Results label), outputs of the PhD project could lead to misuse in the future. Similarly, research outcomes of this PhD project in the future lead to individuals or groups being negatively impacted, either by malicious action or by accident. For example, if based on findings of this research, a drug is developed that can improve or impair memory function. These may seem like a relatively far-fetched example, but it is precisely the point of this exercise to have a warning label that prevents things like these from happening. Mitigations include ensuring clarity in findings and making the code and data accessible for future reference, facilitating corrections if necessary. Additionally, comprehensive documentation should be provided to clarify usage and minimize the potential for misuse.

##### Difficult to Understand - High relevance.

This project includes niche topics such as knowledge of specific postsynaptic protein interactions, and uses specialized software and programming languages for modelling these molecular interactions. Therefore, regarding the project, this label covers two main types of difficulties: firstly, communicating a complicated subject matter, for example communication of the scientific terms to people who are unfamiliar with the research. Secondly, creating transparent research methods and documentation to explain how the models can be run and reproduced.

In order to mitigate these difficulties, an important part of the PhD project has been to make sure biological interactions and their relevance are easily understandable and accessible. Models have been created in accordance with FAIR principles[11], so they can be understood, used and extended by others.

##### May Cause Direct Harm - Medium relevance.

At the moment, causing direct harm to individuals or groups is unlikely due to the computational nature of the research. However, there is potential for animal research based on the results of this work, which may be likely to cause harm to them. Given the complexity of this issue, this is discussed under the newly proposed label Proposed new label: Involves Animal Research below. Furthermore, future studies relating directly to humans may cause direct harm if misused (see Danger of Misuse label).

As a safety precaution, regular reassessment of this work during the project and after its completion is essential, especially if applications become more diverse. In the discussion section we address the questions of who should be responsible for this reassessment and how often it should be done. Additionally, mitigations for future direct harm on animals can include reassessing and applying the principles of the 3Rs (Replacement, Reduction and Refinement) in animal research [119].

##### Risk to Privacy - Low relevance.

Considering the project’s focus on simulations without direct involvement of personal data, the authors do not think there is currently high risk to privacy. Nevertheless, this label will gain relevance if personal data becomes part of the research in the future. For instance, if future research based on this aimed to model specific genetic variations or responses to certain stimuli that are tied to individual human subjects, this could introduce considerations related to privacy, requiring careful ethical handling of sensitive information.

##### Automates Decision‑Making - Low relevance.

While the current focus of the project involves providing insights and informing decisions rather than directly automating them, the machine-readable nature of the work opens up possibilities for future integration into automated decision-making systems. For example, as advancements in technology and artificial intelligence progress, the models and insights generated by this project could potentially be incorporated into automated pipelines for decision-making in neuroscience or related fields. This introduces a future risk where the outputs of the project might play a role in automated processes, emphasizing the importance of anticipating and addressing potential ethical implications as technology evolves.

##### Lacks Informed Consent - Medium relevance.

Given that the current research primarily involves computational simulations and modelling of molecular interactions, the notion of lacking informed consent is less applicable at the moment since there are no human subjects. However, when considering this research is heavily based on data that came from animals in experimental studies, the relevance of informed consent becomes more pronounced. Either way, future safety precautions could involve a thorough assessment of whether to incorporate data from studies lacking informed consent. Additionally, it is essential to explicitly disclose the usage of data originating from studies without consent, providing clear justifications for such usage.

#### Additional step: Proposal of New Labels

In addition to the current Data Hazard labels (as of version 1.0), we propose two further labels which we believe are relevant to this project, and could also be regularly applied to broader research in the future.

##### Proposed new label: Involves Animal Research

Neuroscience, as a field, heavily relies on animal models to explore the complexities of the brain and neurological processes. This involvement raises ethical and methodological concerns that warrant explicit recognition, for instance relating to the translatability of animal research to human health Marshall et al. [120] and about the direct harm caused (see discussion above). Having a label like this adds a layer of ethical and methodological awareness within the neuroscientific Data Hazards framework, emphasizing the need for conscientious and responsible conduct in studies that incorporate animal subjects. Similar to a flammable chemical hazard label, a specific label for “animal research” functions as a warning for researchers,and like the other labels in this framework, it prompts people to consider potential mitigation strategies. One such strategy may involve reducing further animal exploitation.

**High relevance.** This label is relevant for this PhD project and is in close relationship with Reinforces Existing Biases. As laid out in the PhD project’s scope above, data used has come directly or indirectly from animals. Underlying the widespread use of animal experimentation is the assumption that it is fair to use (certain) animals for the purpose of improving human health. These assumptions can be seen as examples of how the research reinforces a speciesist bias, by using data that has been created through a speciesist lens to begin with [121]. Although this PhD project does not involve animal research directly, it emphasizes where data has come from and calls for future researchers to reflect and consider alternative research avenues less reliant on animal subjects where possible (see, for example Ingber [122]). Mitigation strategies for speciesism involve reevaluating current animal research practices and fostering interdisciplinary, inclusive teams that scrutinize classification criteria to uphold fairness, inclusivity, and prevent the perpetuation of biases.

##### Proposed new label: Potential of Faulty Results

This label highlights the possibility of errors during research, which may lead to faulty results. While scientific rigor ideally fosters unbiased, systematic, methodical, and accurate reporting, the acknowledgement of potential errors safeguards and reminds future researchers to be vigilant in understanding the datasets and analyses used before they apply them in further work. Faulty results can manifest in many ways, such as (but not restricted to) inconsistencies in graph outputs; erroneous statistical reporting [123],errors in the use of programming languages due to typos or misinterpretation of function documentation; misreporting of methods in wet-lab research. Mitigations for this label may not always be explicit, but rather be implicit in the form of good research practices [124–126] and scientific rigour [127]. Addressing potential sources of error enhances the reproducibility of findings and minimizes the risk of wasted time and resources due to futile replication attempts.

**High relevance.** This label is highly relevant to this PhD project, as it would likely be for many research projects. For instance, coding errors, data anomalies, or misinterpretations in this project could lead to flawed conclusions about the interaction between the “memory molecules” of interest. Mitigation strategies for this label are embedded throughout the research cycle and include thorough code reviews, validation of results for logical coherence, and the implementation of sanity checks to ensure data accuracy and reliability (see related hazard labels and their mitigations such as Danger of Misuse and Difficult to Understand).

#### Method 2: How do the Labels Apply Through the PhD Project Research Life Cycle?

In Method 1 the labels’ relevance is described more generally in regards to the whole project. Here in Method 2, we identify how labels can have higher or lower relevance during different stages of the project’s life cycle (Table 2). For example, the “Reinforces Existing Biases” label is seen as more relevant during the design and data collection stages, as the data collected carries various systemic biases (see Reinforces Existing Biases and Discussion), but as mitigations and safety precautions are put into action, when reporting the project’s outcomes, we expect the risk to have decreased and therefore the relevance of the label too. Similarly, the “High Environmental Impact” label is most relevant during the data analysis phase when resources are heavily used, but it becomes less pertinent when reporting results due to the lesser environmental impact associated with reporting. The label “Lacks Community Involvement” is seen to have low relevance throughout all stages of this PhD, as the project has a big emphasis on including the scientific community by having an open-science approach and applying FAIR principles. For the “Danger of Misuse” label, the highest relevance would apply to the data analysis and reporting stages, as these are the times when people could misuse data due to error in the analysis or for malintent (for more details see Danger of Misuse). “Difficult to Understand” is seen as highly relevant at all stages as the topics studied are niche and multidisciplinary, hence requiring previous specific knowledge to understand. As described in Method 1, currently, the computational nature of the PhD’s research makes it unlikely to directly harm individuals or groups, so the “May Cause Direct Harm” label becomes more relevant with how results are reported and future work. The new label “Involves Animal Research” highlights that data used for this PhD project originates from animal experimentation, emphasizing the need to consider methods and future experiments related to this work. Labels such as “Ranks or Classifies People”, “Automates Decision-Making” or “Risk to Privacy” are less relevant throughout, since this PhD project does not deal with personal information, but as described in Method 2 future work should consider precaution measures as necessary. Finally, “Potential of Faulty Results” can be applicable at all stages of this PhD, as on the one hand, there is potential of using already faulty results, but also the possibility of outputting faulty results from this work too.

**Table 2:** Data Hazard labels and their relevance at different stages of the research life cycle. (+ +): the label has high relevance at this stage, ( +): the label has moderate relevance at this stage, (-): the label has low relevance at this stage. Mitigations are described in Method 1

Data Hazard Label	Design	Data Collection	Data Analysis	Reporting	Mitigations/Safety Precautions
General Data Hazard	( +)	( +)	( +)	( +)	see General Data Hazard
Reinforces Existing Biases	(+ +)	(+ +)	( +)	( +)	see Reinforces Existing Biases
Ranks or Classifies People	(-)	(-)	(-)	( +)	see Ranks or Classifies People
High Environmental Impact	( +)	( +)	(+ +)	( +)	see High Environmental Impact
Lacks Community Involvement	(-)	(-)	(-)	(-)	see Lacks Community Involvement
Danger of Misuse	(-)	(-)	( +)	( +)	see Danger of Misuse
Difficult to Understand	(+ +)	(+ +)	(+ +)	(+ +)	see Difficult to Understand
May Cause Direct Harm	(-)	(-)	( +)	( +)	see May Cause Direct Harm
Risk to Privacy	(-)	(-)	(-)	(-)	see Risk to Privacy
Automates Decision-Making	(-)	(-)	(-)	(-)	see Automates Decision-Making
Lacks Informed Consent	( +)	( +)	(-)	(-)	see Lacks Informed Consent
Involves Animal Research	( +)	( +)	(-)	( +)	see Proposed new label: Involves Animal Research
Potential of Faulty Result	(+ +)	(+ +)	(+ +)	(+ +)	see Proposed new label: Potential of Faulty Results

## Discussion

As neuroscience continues to broaden, new ethical considerations arise which require new ethical approaches. As a general ethical approach to any data-related project, the Data Hazard framework has the potential of being applied to many disciplines in the life sciences; this includes both disciplines where clear ethical issues have been already highlighted, such as synthetic biology [13], and other areas that may not deal directly with ethically obvious issues, such as computer simulations and mathematical models using previously collected data. In the latter context, the dangers of a project not involving humans or animals directly may not be immediately apparent. This paper presents a PhD as a case study of a computational neuroscience project, using computer simulations of interactions between CaMKII and NMDARs. We use this to highlight a topic not commonly associated with ethical evaluation, and how its ethical risks can be discussed and addressed with Data Hazards labels. We have proposed different mitigation strategies for issues which we can tackle now, and raise awareness for work to be done in the future. We have also proposed two new labels: “Involves Animal Research”, and “Potential of Faulty Result”, to extend the Data Hazard’s relevance to neuroscience.

Throughout the ethical evaluation of Data Hazard labels applicable to the PhD case study described, we have seen how some labels are more relevant than others. These classifications are dynamic and subject to change over time. When applying hazard labels to the PhD project, higher-relevance labels call for more immediate application of mitigation strategies. Conversely, less relevant labels prompt considerations for future research, acknowledging potential risks that may arise in subsequent work. Regarding future research, there is also a philosophical and political question of who is responsible for future research risks [128, 129]. Should the researcher identify possible future dangers and mitigations, or should future researchers consider mitigation strategies once they become tangible. When labelling one’s own work with potential future privacy risks, automating decision-making or dealing with informed consent, the labels act as a warning message that future research should consider these implications, without assigning responsibility to anyone. While the Data Hazards toolkit facilitates individual accountability, addressing systemic issues may be more effective at the institutional level. In this context, the Data Hazard labels act as initial warnings, encouraging a proactive examination of the ethical dimensions of scientific research, not as a tool that will address or solve the issues at hand.

Additionally, the process of applying Data Hazard labels and determining their relevance inherently involves a degree of subjectivity, as interpretations will inevitably vary based on individuals’ ethical beliefs, cultural backgrounds, upbringing, religious views, and other personal factors (see questions of positionality under Reinforces Existing Biases); researchers from different backgrounds might have different willingness and/or awareness to address specific ethical issues. Unlike chemical hazard labels, the labels used here address moral dilemmas and philosophical questions that do not lend themselves to rigid categorization. The framework is intentionally designed to be flexible, encouraging dialogue and reflection on these complex issues. Notably, even among the authors of this paper, there were disagreements and debates regarding the applicability and priority of different labels throughout. To illustrate one example, the authors of this paper debated over the use of an ableist label (see Reinforces Existing Biases) for the project case study. Does frequent use of this label dilute its significance, or does it serve to bring critical awareness to the uncomfortable reality that we may be operating within an oppressive system, failing to recognise and question its underlying ableist assumptions? Similar questions were raised for other labels too. We do not intend to provide a definitive answer to this question, nor is that the purpose of employing Data Hazard labels. Rather, the objective is to bring these questions to the forefront, where they might otherwise remain unexamined. This illustrates the value of Data Hazard labels in stimulating critical discussions and, ideally, encouraging a more reflective and conscientious scientific practice.

Researchers often have to engage with multiple ethical review processes at different levels during the course of a project. These can include drafting research ethics statements for grant proposals, seeking local ethical approvals for projects, or obtaining clearance for individual experimental procedures. When it comes to integrating and applying the Data Hazards toolkit, the labels can be used as a complementary resource to assist researchers in meaningfully fulfilling these legal and institutional requirements. Alternatively, the toolkit can be applied more systematically, generating a document for upload to a public repository, which can then be linked in an ethics board application or included as an appendix to a publication (see table example to use in Supplementary Materials).

Additionally, interactions between researchers and diverse stakeholders highlight the importance of community involvement The definition of “community” and the scope of their involvement will vary with context (see Lacks Community Involvement). For instance, community-driven frameworks such as *TTW: The Turing Way* and *FORRT: Framework for Open and Reproducible Research Training* engage broader scientific communities across varied disciplines [15, 130–132], whereas more specialized frameworks focus on directly involving specific communities affected by research [116]. The Data Hazards labels align with and are well-suited for integration into these community frameworks. In fact, it is already included as a chapter in The Turing Way and could complement more specialized community assessment practices [133] by fostering an interdisciplinary vocabulary to discuss and reflect on ethical risks within specific fields [118].

A potential drawback of the Data Hazards project is the balance between specificity and generality. As the project aims to offer a broad ethical framework applicable across diverse scientific domains, including neuroscience, there is a risk of it being perceived as too general for some contexts. The intricacies of neuroscientific research, with its diverse methodologies and subject matter, make it challenging to create a one-size-fits-all conceptual framework.

Despite this, the framework serves as a valuable starting point. It provides researchers with a common language for discussing risks and ethical considerations, thereby fostering interdisciplinary collaboration. A general toolkit that can be honed and applied to specific fields may be more easily applicable than the other way around. The challenges identified are inherent to the ongoing development of the toolkit, which is not a static document but rather a community-driven, living project. Multiple versions and iterations reflect the commitment to refining and expanding the framework to address emerging challenges and ensure its relevance in the ever-changing landscape of neuroscientific research.

There is also the challenge of impact. Labelling something as hazardous does not change its hazardous properties or reduce its inherent risk. So, what is the impact of labelling something, will it change the outcome of the research? If we consider the parallel of chemical hazard labels, warning the user that a substance is ‘flammable’ does not change its flammability, but instead allows the user to make choices based on this knowledge. We know to handle it with care and take the necessary precautions. This applies to the Data Hazards labels too, where if we identify the risks, we can act accordingly. Moreover, the action of labelling an activity encourages reflection and conscious awareness, which in itself has been shown to have benefits [134–136].

## Concluding Thoughts

Here we have presented the Data Hazards framework as a tool to consider potential risks and consequent mitigations for a case study in neuroscience. The Data Hazards framework is a tool for considering the wide implications of a research programme and reflecting on nuanced and complex topics such as bias, ethics and philosophy in the ever-growing field of neuroscience. This framework is designed to flexibly accommodate new use cases and remain extendable to adapt to different disciplines and their emerging ethical concerns. As it is an open-source, community-driven project, this allows it to develop in line with grassroots concerns, and can work in combination with ‘top down’ approaches. Overall, the Data Hazards Framework is a conceptual framework that sets a foundational vocabulary for reflections that will enable neuroscience to advance towards more ethical and inclusive scientific research.

## Supplementary Information

Below is the link to the electronic supplementary material.

Supplementary file1 (DOCX 19 KB)
