# Supplementary material for: Data Hazards as An Ethical Toolkit for Neuroscience
Source: Neuroethics. 2025 Feb 19;18(1):15. doi: 10.1007/s12152-024-09580-3 (PMC11835915; doi:10.1007/s12152-024-09580-3)
Supplement: Supplementary file 1 — Supplementary file1 (DOCX 19 KB) [file 12152_2024_9580_MOESM1_ESM.docx]

# Template for applying Data Hazards to the research life cycle

Please use the table template below to fit your own project.

You may choose to use the following shorthand illustrated in Table 2. (++): the label has high relevance at this stage, (+): the label has moderate relevance at this stage, (-): the label has low relevance at this stage.

| **Data Hazard Label** | **Design** | **Data Collection** | **Data Analysis** | **Reporting** | **Mitigations/Safety Pre-cautions** |
| --- | --- | --- | --- | --- | --- |
| General Data Hazard |  |  |  |  |  |
| Reinforces Existing Biases |  |  |  |  |  |
| Ranks or Classifies People |  |  |  |  |  |
| High Environmental Impact |  |  |  |  |  |
| Lacks Community Involvement |  |  |  |  |  |
| Danger of Misuse |  |  |  |  |  |
| Difficult to Understand |  |  |  |  |  |
| May Cause Direct Harm |  |  |  |  |  |
| Risk to Privacy |  |  |  |  |  |
| Automates Decision-Making |  |  |  |  |  |
| Lacks Informed Consent |  |  |  |  |  |
| Involves Animal Research |  |  |  |  |  |
| Potential of Faulty Result |  |  |  |  |  |
